# Usefulness of waist-to-height ratio in screening incident metabolic syndrome among Japanese community-dwelling elderly individuals

**DOI:** 10.1371/journal.pone.0216069

**Published:** 2019-04-29

**Authors:** Ryuichi Kawamoto, Asuka Kikuchi, Taichi Akase, Daisuke Ninomiya, Teru Kumagi

**Affiliations:** 1 Department of Community Medicine, Ehime University Graduate School of Medicine, Ehime, Japan; 2 Department of Internal Medicine, Seiyo Municipal Nomura Hospital, Ehime, Japan; International University of Health and Welfare, School of Medicine, JAPAN

## Abstract

This study examined a range of anthropometric indices and their relationships with metabolic syndrome (MetS). Despite recommendations that central obesity assessment should be employed as a marker of metabolic health, there is no consensus regarding the protocol for measurement. The present study included 720 men aged 71 ± 8 years and 919 women aged 71 ± 7 years from a rural village. We examined the relationship between anthropometric indices {e.g., body mass index (BMI), waist-to-height ratio (WHtR), waist-to-hip ratio (WHpR)}, and MetS based on the modified criteria of the National Cholesterol Education Program’s Adult Treatment Panel (NCEP-ATP) III report in a cross-sectional (N = 1,639) and cohort (N = 377) data. A receiver operating curve (ROC) analysis was performed to determine the optimal cut-off value and best discriminatory value of each of these anthropometric indices to predict MetS. In the cross-sectional study, WHtR as well as BMI and WHpR showed significantly predictive abilities for MetS in both genders; and WHtR showed the strongest predictive ability for the presence of MetS. Also in the cohort study, WHtR as well as BMI and WHpR showed significantly predictive abilities for incident MetS in both genders, and in men WHtR showed the strongest predictive ability for incident MetS, but in women BMI showed the strongest predictive ability. In the cross-sectional study, the optimal WHtR cutoff values were 0.52 (sensitivity, 71.0%; specificity, 77.9%) for men and 0.53 (sensitivity, 79.8%; specificity, 75.7%) for women. In the cohort study, the optimal WHtR values were 0.50 (sensitivity, 60.7%; specificity, 73.2%) for men and 0.50 (sensitivity, 75.0%; specificity, 56.1%) for women. Increased WHtR was significantly and independently associated with prevalence of MetS in both genders. These results suggest that WHtR is a useful screening tool for determining metabolic risk in Japanese elderly community dwelling individuals.

## Introduction

The underlying mechanism of metabolic syndrome (MetS), or a clustering of cardiovascular risk factors, such as hypertension, glucose intolerance, hypertriglyceridemia, and low high-density lipoprotein cholesterol (HDL-C) levels, is insulin resistance, which is also known as a pre-disease state that leads to an increased risk of cardiovascular disease (CVD) [1], [2], and type 2 diabetes [3], [4]. The incidence of MetS is increasing worldwide with the continuous increase in obesity prevalence [5]. Obesity is the most important underlying cause of insulin resistance, which has also been suggested as a pathogenetic mechanism of abdominal obesity because visceral fat plays an important role in lipid metabolism and insulin sensitivity [6], [7], [8], [9]. Various obesity-related anthropometric indices, such as body mass index (BMI), waist circumference (WC), waist-to-height ratio (WHtR), and waist-to-hip ratio (WHpR), have been used to predict incident MetS in epidemiological studies [10], [11], [12], [13], [14]. BMI is a measurement of body fat by height and weight, while WC reflects abdominal obesity. WHtR and WHpR further reflect the fat distribution by WC, and all three indices are considered to be specific options to evaluate abdominal fat.

To address this hypothesis, we investigated the relationship between baseline visceral obesity indices and potential risk factors such as age, smoking status, drinking status, exercise habits, presence of CVD, low-density lipoprotein cholesterol (LDL-C), serum uric acid (SUA), estimated glomerular filtration ratio (eGFR), and incident MetS using prospective cohort data from community-dwelling elderly individuals.

## Materials & methods

### Subjects

The subjects of this study population were recruited from the Nomura Health and Welfare Center in a rural town in Ehime prefecture of Japan through annual health checkup process closely related to the area (17). This study was started in 2014, and included 1639 community-dwelling participants aged 55–95 years. Follow-up assessment cycles are performed every three years.

**Fig 1:**
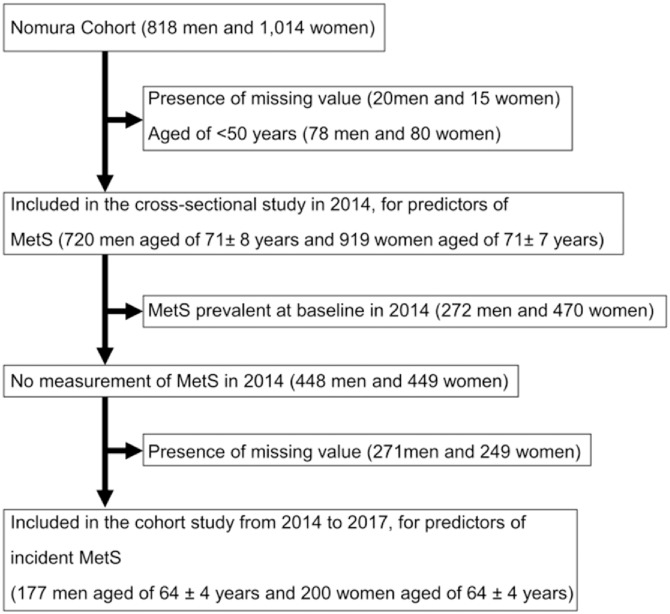
Flowchart. For the cross-sectional analyses, data of the 2014 cycle (n = 1,639) that were used in this cycle were measured. For the longitudinal analyses, only participants in whom MetS was not prevalent at baseline in 2014 were included in the longitudinal analyses (n = 377).

In the present study, we included data from the assessment cycles of 2014 and 2017. Blood samples were only obtained from respondents who participated in the medical interview at baseline. For the cross-sectional analyses, data of the 2014 cycle (n = 1639) were used as all five components of MetS were measured in this cycle. For the longitudinal analyses, a sub-cohort of the 2014 cycle was used including only participants in whom MetS was not prevalent at baseline in 2014 (n = 377). Fig 1 shows a flowchart of the inclusion of participants.

This study complies with the Declaration of Helsinki, written informed consent was obtained from each subject, and the study was approved by the Ehime University Medical School Ethics Committee. All procedures performed in the study involving human participants were in accordance with the ethical standards of the institutional research committee in which the study was conducted. (IRB Approval number: 1402009).

### Evaluation of confounding factors

Information on demographic characteristics and risk factors was collected using clinical files. Body mass index (BMI) was calculated by dividing weight (kilograms) by the square of height (meters). WHtR was calculated as WC (cm)/height (cm). WHpR was calculated as WC (cm)/hip circumference (cm). Other characteristics such as exercise, smoking habit, alcohol consumption, and medication, were investigated by individual interviews conducted using a structured questionnaire. Smoking habit was defined as the number of cigarette packs per day multiplied by the pack years (pack year), and participants were classified into never smokers, past smokers, light smokers (<30 pack year), and heavy smokers (≥30 pack year) [15]. Daily drinking status was measured using the Japanese alcoholic beverage unit equivalent to 22.9 g of ethanol, and the participants were classified into never drinkers, occasional drinkers (<1 unit/day), daily light drinkers (<2 unit /day), and daily heavy drinkers (≥2 unit/day) [16]. We measured blood pressure (BP) on the right upper arm of the subjects with an appropriate-sized cuff in the sedentary position using an automatic oscillometric blood pressure recorder after having rested for at least 5 min. For analysis, the mean of two consecutive measurements was used.

For all these individuals, triglycerides (TG), HDL-C, low-density lipoprotein cholesterol (LDL-C), hemoglobin A1c (HbA1c), serum uric acid (SUA), and creatinine (Cr) were measured during an overnight fast of over 11 hours. eGFR was calculated using CKD-EPI equations modified by the coefficient of Japan (eGFR_CKDEPI_): Male, Cr ≤0.9 mg/dl, 141 × (*Cr*/0.9) –^0.411^ × 0.993 ^*age*^ × 0.813; Cr >0.9 mg/dl, 141 × (*Cr*/0.9) –^1.209^ × 0.993 ^*age*^ × 0.813; Female, Cr ≤0.7 mg/dl, 144 × (*Cr*/0.7) –^0.329^ × 0.993 ^*age*^ × 0.813; Cr >0.7 mg/dl, 144 × (*Cr*/0.7) –^1.209^ × 0.993 ^*age*^ × 0.813 (18).

### Criteria for clinical diagnosis of MetS

Based on the modified criteria of the National Cholesterol Education Program’s Adult Treatment Panel (NCEP-ATP) III report [17], MetS was defined as subjects having at least three of the following five conditions: 1) abdominal obesity of waist circumference ≧85 cm for men and ≧80 cm for women based on the adjusted waist circumference criteria in Japan [18]; 2) high BP with a systolic blood pressure (SBP) ≥130 mmHg and/or diastolic blood pressure (DBP) ≥85 mmHg, and/or drug treatment for elevated blood pressure; 3) hypertriglyceridemia with a TG level ≥150 mg/dL; 4) low HDL cholesterolemia with a HDL-C <40 mg/dL for men and <50 mg/dL for women, and/or drug treatment for dyslipidemia; and 5) high fasting glucose with a HbA1c ≥5.6% (comparable with fasting plasma glucose (FPG) level ≥100 mg/dL [19] because FPG was not measured in this study) and/or drug treatment for elevated blood sugar.

### Statistics

Unless otherwise specified, data are presented as the mean ± standard deviation (SD) and for parameters with non-normal distributions (i.e., TG, HbA1c) data are shown as median (interquartile range) values. For all analyses, parameters with non-normal distributions were used after log-transformation. Statistical analysis was performed using IBM SPSS Statistics Version 21 (Statistical Package for Social Science Japan, Inc., Tokyo, Japan). Subjects were divided into two groups based on gender and differences among the groups were analyzed by Student’s t-test for continuous variables or the χ^2^ -test for categorical variables. Multiple logistic linear regression analysis was used to evaluate the contribution of the baseline WHtR and confounding factors (i.e., gender, age, exercise habit, smoking habits, alcohol consumption, and prevalence of CVD, LDL-C, SUA, and eGFR) for prevalence of MetS in the cross-sectional study and incidence of MetS in the cohort study. In addition, areas under the receiver operating characteristic (ROC) curves were determined for each variable to identify the predictors of MetS. An ROC curve is a plot of sensitivity (true positive) versus 1–specificity (false positive) for each potential marker tested. Areas under the ROC curves are provided with standard errors. The area under the ROC curve is a summary of the overall diagnostic accuracy of the test. The best marker has an ROC curve shifted to the left with area under the curve close to unity. Predictive values were calculated as *sensitivity*/{*sensitivity*+(1 − *specificity*)} (positive predictive value) and *specificity*/{(1 − *sensitivity*)+*specificity*} (negative predictive value). To determine the optimal cutoffs for the MetS, the Youden index (*sensitivity* + *specificity* − 1) was calculated, and the corresponding value for the maximum of the Youden index was considered as the optimal cutoff point. A *p*-value <0.05 was considered significant.

## Results

### Baseline characteristics of study subjects categorized by gender

Baseline characteristics of the subjects categorized by gender are illustrated in Table 1. The study included 720 men aged 71 ± 8 (range, 55–95) years and 919 women aged 71 ± 7 (range, 55–90) years. BMI, WC, WHpR, smoking status, drinking status, prevalence of CVD, DBP, TG, HbA1c, presence of antidiabetic medication, and SUA were significantly higher in men, but WHtR, HDL-C, LDL-C, presence of antidyslipidemic medication, and eGFR were significantly lower. There were no differences in age, exercise habits, SBP, and presence of antihypertensive medication. In our study, prevalence of MetS was 37.8% in men and 51.1% in women, and mean (± SD) number of its component was 2.3 (± 1.1) in men and 2.6 (± 1.2) in women.

**Table 1:** Baseline characteristics of study subjects.

Baseline Characteristics N = 1,639	Men N = 720	Women N = 919	*P*-value*
Age (years)	71 ± 8	71 ± 7	0.602
Body mass index (kg/m^2^)	23.1 ± 2.9	22.6 ± 3.2	**<0.001**
Waist circumference (cm)	82.4 ± 8.1	80.5 ± 9.0	**<0.001**
Waist/height ratio	0.51 ± 0.05	0.54 ± 0.06	**<0.001**
Waist/hip ratio	0.90 ± 0.06	0.89 ± 0.06	**<0.001**
Smoking habit (never/past/light/heavy (%))	41.7/40.6/4.3/13.5	96.8/2.1/0.7/0.4	**<0.001**
Drinking Status (never/occasional/light/heavy (%))	24.7/22.1/16.8/36.4	71.6/22.1/4.6/1.7	**<0.001**
Exercise habits (%)	36.9	38.6	0.505
Cardiovascular disease (%)	10.3	4.4	**<0.001**
Systolic blood pressure (mmHg)	137 ± 17	137 ± 18	0.714
Diastolic blood pressure (mmHg)	80 ± 10	77 ± 10	**<0.001**
Antihypertensive medication (%)	48.1	45.3	0.272
Triglycerides (mg/dl)	90 (68–131)	87 (65–117)	**<0.001**
HDL cholesterol (mg/dl)	62 ± 16	68 ± 17	**<0.001**
LDL cholesterol (mg/dl)	114 ± 28	124 ± 29	**<0.001**
Antidyslipidemic medication (%)	14.0	30.0	**<0.001**
Hemoglobin A 1c (%)	5.7 (5.4–6.0)	5.7 (5.5–5.9)	**0.036**
Antidiabetic medication (%)	13.6	5.5	**<0.001**
Serum uric acid (mg/dL)	6.0 ± 1.3	4.7 ± 1.1	**<0.001**
Estimated GFR (ml/min/1.73 m^2^/year)	69.4 ± 12.1	71.8 ± 10.8	**<0.001**
Number of metabolic syndrome component	2.3 ± 1.1	2.6 ± 1.2	**<0.001**
Metabolic syndrome (%)	37.8	51.1	**<0.001**

HDL, high-density lipoprotein; LDL, low-density lipoprotein; GFR glomerular filtration ratio. Data presented are mean ± standard deviation. Data for triglycerides and HemoglobinA1c is skewed, and presented as median (interquartile range) values.

* *P*-value: Student’s t-test for the continuous variables or the χ^2^ -test for the categorical variables. Bold values indicate significance (*p*<0.05).

### Results of the ROC curve analysis to identify optimal obesity indices to discriminate subjects with MetS in the cross-sectional and cohort studies

**Fig 2:**
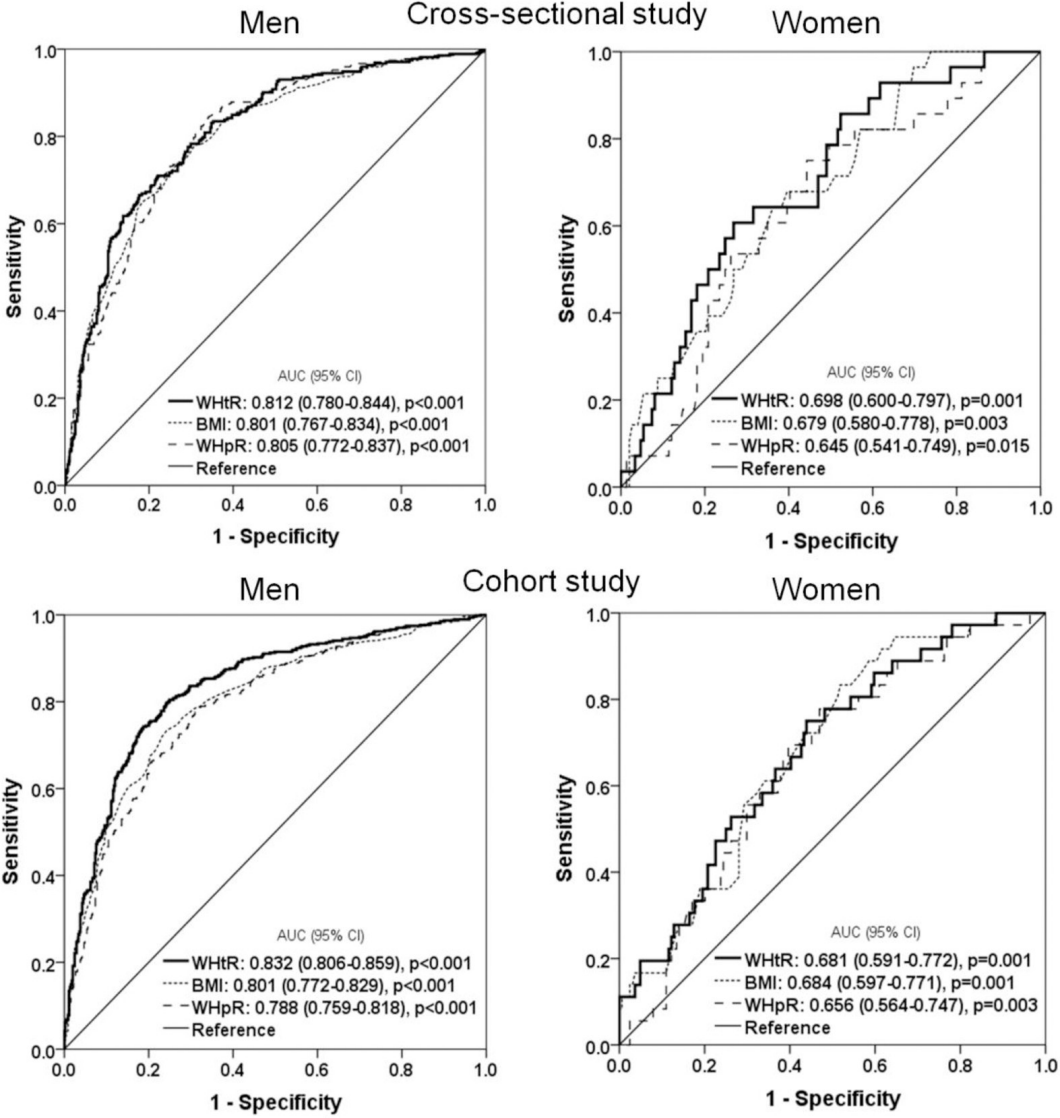
Area under the receiver operating curve (AUC) values (95% CI) for selected obesity measurements to discriminate subjects with metabolic syndrome in the cross-sectional and cohort study.

Fig 2 shows the AUC for WHtR, BMI, and WHpR for each MetS in both genders using ROC analyses. In the cross-sectional study, WHtR as well as BMI and WHpR showed significantly predictive ability for MetS in both genders, with WHtR showing the strongest predictive ability. Also in the cohort study, WHtR as well as BMI and WHpR showed a significantly high predictive ability for incident MetS in both genders. In men WHtR showed the strongest predictive ability for incident MetS, but in women BMI showed the strongest predictive ability.

### Non-adjusted odds ratios and 95% CI for MetS and its components by quartile of WHtR in the cross-sectional and cohort study

The univariate effect of WHtR on MetS and its components is presented in Table 2. In the cross-sectional study, WHtR was significantly associated with presence of MetS and all of its components in both genders. In the cohort study, WHtR was significantly and independently associated with incident MetS and central obesity in both genders.

**Table 2:** Non-adjusted odds ratios and 95% CI for metabolic syndrome and its components of subjects according to baseline waist to height ratio in the cross-sectional and cohort studies.

Cross-sectional study N = 1,639	Men N = 720			Women N = 919		
	Yes/No	Odds ratio (95% CI)	*P*-value*	Yes/No	Odds ratio (95% CI)	*P*-value
Metabolic syndrome	272/448	3.62 (2.95–4.43)	**<0.001**	470/449	3.46 (2.93–4.08)	**<0.001**
Central obesity	254/466	15.8 (10.6–23.3)	**<0.001**	471/448	19.9 (13.4–29.5)	**<0.001**
Elevated blood pressure	572/148	1.62 (1.34–1.97)	**<0.001**	716/203	1.66 (1.43–1.91)	**<0.001**
Elevated triglycerides	128/592	1.62 (1.35–1.95)	**<0.001**	112/807	1.87 (1.50–2.33)	**<0.001**
Lowering HDL cholesterolemia	139/581	1.62(1.35–1.95)	**<0.001**	349/570	1.54 (1.35–1.75)	**<0.001**
Elevated hemoglobin A 1c	527/193	1.24 (1.05–1.47)	**0.011**	704/215	1.17 (1.02–1.34)	**0.026**
**Cohort study N = 377**	**Men N = 177**			**Women N = 200**		
Metabolic syndrome	28/149	1.99 (1.21–3.28)	**0.007**	36/164	2.02 (1.34–3.06)	**0.001**
Central obesity	41/136	5.38 (2.96–9.77)	**<0.001**	54/146	5.51 (3.29–9.23)	**<0.001**
Elevated blood pressure	125/52	1.18 (0.76–1.82)	0.459	117/83	1.01 (0.74–1.39)	0.956
Elevated triglycerides	21/156	1.03 (0.57–1.86)	0.930	13/187	1.03 (0.55–1.93)	0.933
Lowering HDL cholesterolemia	16/161	0.75 (0.36–1.57)	0.450	31/169	1.50 (0.98–2.28)	0.061
Elevated hemoglobin A 1c	107/70	0.89 (0.60–1.31)	0.551	129/71	0.82 (0.59–1.13)	0.219

CI, confidence interval.

*Bold values indicate significance (*p*<0.05).

### Multivariate-adjusted odds ratios and 95% CI for MetS and its components by quartile of WHtR in the cross-sectional and cohort studies

To further investigate whether WHtR can explain MetS and its components independently of other confounding factors, a multiple logistic regression analysis using MetS and its components as dependent variables and various confounding factors (e.g., age, smoking status, drinking status, exercise habits, presence of CVD, LDL-C, SUA, and eGFR) as explanatory variables was performed with subjects categorized by gender (Table 3). In both the cross-sectional and cohort studies, increased WHtR showed an increasing trend with increased prevalence of MetS in both genders.

**Table 3:** Multivariate-adjusted odds ratios and 95% CI for metabolic syndrome and its components of subjects according to baseline waist to height ratio in the cross-sectional and cohort studies.

Cross-sectional study N = 1,639	Men N = 720			Women N = 919		
	Yes/No	Odds ratio (95% CI)	*P*-value*	Yes/No	Odds ratio (95% CI)	*P*-value*
Metabolic syndrome	272/448	3.82 (3.08–4.72)	**<0.001**	470/449	3.33 (2.81–3.96)	**<0.001**
Central obesity	254/466	19.5 (12.6–30.1)	**<0.001**	471/448	31.0 (19.5–49.2)	**<0.001**
Elevated blood pressure	572/148	1.57 (1.28–1.93)	**<0.001**	716/203	1.43 (1.23–1.68)	**<0.001**
Elevated triglycerides	128/592	1.59 (1.30–1.95)	**<0.001**	112/807	1.75 (1.38–2.21)	**<0.001**
Lowering HDL cholesterolemia	139/581	1.70 (1.40–2.07)	**<0.001**	349/570	1.48 (1.28–1.70)	**<0.001**
Elevated hemoglobin A1c	527/193	1.24 (1.04–1.47)	**0.016**	704/215	1.10 (0.95–1.28)	0.200
**Cohort study N = 377**	**Men N = 177**			**Women N = 200**		
Metabolic syndrome	28/149	1.94 (1.14–3.32)	**0.015**	36/164	1.93 (1.23–3.03)	**0.004**
Central obesity	41/136	5.75 (3.05–10.8)	**<0.001**	54/146	8.28 (4.28–16.0)	**<0.001**
Elevated blood pressure	125/52	1.07 (0.68–1.69)	0.769	117/83	0.88 (0.62–1.24)	0.467
Elevated triglycerides	21/156	1.07 (0.55–1.90)	0.960	13/187	1.09 (0.55–2.19)	0.803
Lowering HDL cholesterolemia	16/161	1.07 (0.48–2.37)	0.869	31/169	1.27 (0.82–1.96)	0.292
Elevated hemoglobin A 1c	107/70	0.90 (0.59–1.40)	0.648	129/71	0.83 (0.58–1.17)	0.280

*Multivariate-adjusted for age, smoking status, drinking status, exercise habits, presence of cardiovascular disease, low-density lipoprotein cholesterol, serum uric acid, and estimated GFR. Bold values indicate significance (*p*<0.05).

### Best cutoff values of WHtR to predict MetS in the cross-sectional and cohort studies

In the cross-sectional study, the optimal WHtR cutoff values for predicting MetS according to WHtR were 0.52 (sensitivity, 71.0%; specificity, 77.9%) for men and 0.53 (sensitivity, 79.8%; specificity, 75.7%) for women (Table 4). In the cohort study, the optimal WHtR values were 0.50 (sensitivity, 60.7%; specificity, 73.2%) for men and 0.50 (sensitivity, 75.0%; specificity, 56.1%) for women.

**Table 4:** Best cutoff values of baseline waist to height ratio to predict metabolic syndrome in the cross-sectional and cohort studies.

	AUC (95% CI)	*P*-value	Cut off value	Sensitivity	specificity	PPV	NPV
**Cross-sectional study N = 1639**							
Men N = 720	0.812 (0.780–0.844)	**<0.001**	0.5185	71.0%	77.9%	76.3%	72.9%
Women N = 919	0.832 (0.806–0.859)	**<0.001**	0.5349	79.8%	75.7%	76.7%	78.9%
**Cohort study N = 377**							
Men N = 177	0.698 (0.600–0.797)	**0.001**	0.4991	60.7%	73.2%	69.4%	63.1%
Women N = 200	0.681 (0.591–0.772)	**0.001**	0.4957	75.0%	56.1%	62.2%	69.2%

AUR, Area under the receiver operating curve; PPV: positive predictive value; NPV: negative predictive value. Bold values indicate significance (*p*<0.05).

## Discussion

In this study where data from the Nomura study of 2014 and 2017 was used, the AUC analyses indicated that WHtR as well as BMI and WHpR had significant predictive ability for MetS in both genders, and that WHtR was significantly and independently associated with the prevalence of MetS in this cross-sectional study as well as the incidence of MetS in this cohort study. The usefulness of this cutoff value as a screening tool for the prediction of MetS was superior to those of BMI and WHpR, which are conventional obesity indices among both genders. This study showed that WHtR might be an appropriate definition from the point of view of knowing the presence and incidence of MetS. To the best of our knowledge, few epidemiologic studies have quantified the relevance between WHtR and incident MetS in Japanese elderly community-dwelling individuals.

This cross-sectional study showed that WHtR as well as BMI and WHpR was useful for predicting MetS, which is consist with Gu et al.’s research [20]. From the AUC analysis, BMI, WC and WHtR were predictive of high metabolic risks in men (0.698, 0.691, and 0.688, respectively), whereas female BMI and WC were similarly predictive of high metabolic risks (0.676 and 0.669) [20]. According to Liu et al. [21], ROC analyses of BMI, WC and WHtR values indicated that the presence of multiple metabolic risk factors can be equally predicted in Chinese adult population, and the AUC values of BMI, WC and WHtR did not differ in men (0.682, 0.661, and 0.651, respectively) and women (0.702, 0.671, and 0.674, respectively). The appropriate cut-off values for BMI, WC and WHtR were 22.9 and 23.3 kg/m^2^, 91.3 cm and 87.1 cm, and 0.51 and 0.53 in men and women, respectively. Zeng et al. [13] demonstrated that the optimal cut-off values to define overweight or obesity in Chinese adults were approximately 24·0 and 23·0 kg/m^2^ for BMI, 85·0 and 75·0 cm for WC, and 0·50 and 0·48 for WHtR for men and women, respectively. Ashwell et al. reported that in a systematic review and meta-analysis, WC improved identification of harmful cardiovascular risk outcomes by 3% compared with BMI, and WHtR improved discrimination by 4 to 5%. Moreover, WHtR was a stronger predictor than WC for hypertension, diabetes, CVD and all outcomes in both genders (*p*<0.005) [22]. The AUC analyses indicated that WHtR may be a more useful global clinical screening tool than WC and has a weighted mean boundary value of 0·5 [23]. In our prospective cohort study, WHtR was a convenient global clinical screening tool with a weighted mean boundary value of 0.50 in both genders.

The mechanisms that lead to increased incidence of MetS in individuals with increased WHtR remain to be clarified. BMI is strongly related to body fat but is not necessarily related to abdominal obesity. WC may accurately reflect the degree of visceral fat, but WC can overestimate or underestimate the risk of CVD as WC does not take into account differences in height [24], [25]. Hsieh et al. showed that people with a prominently large WC may have similar health risks of the above items irrespective of height, but short people have higher health risks than tall people in the moderately large WC population of Japanese men. [24]

Several limitations should be considered in this study. First, our cross-sectional study design does not eliminate the cause and effect on conventional obesity indices and MetS. Second, the measurement of WHtR is based on a single evaluation of the equation, which may introduce a misclassification bias. Third, we could not eliminate the influence that taking medications for hypertension, dyslipidemia, and hyperglycemia has on the present findings. Fourth, as the WC component is included in the MetS definition, the AUC estimate for the WHtR can be disturbed. Fifth, the longitudinal analyses were limited by a smaller sample size and discrepancies in the sequential measurements of the components of MetS in 2014 and 2017. The cohort was slightly younger and healthier compared to participants not included in the longitudinal analyses, this might have caused an underestimation of incident MetS after three years of follow-up. Therefore the demographics and referral source may limit generalizability of the study findings.

### Conclusions

The present study showed that anthropometric indices such as WHtR, BMI, and WHpR are strongly associated with incident MetS among Japanese community-dwelling individuals. The underlying mechanism behind this relationship is unknown, but it seems to be independent of confounding factors such as age, exercise habits, smoking habits, drinking status, prevalence of CVD, LDL-C, SUA or eGFR. Thus, WHtR might be an important marker for the assessment of risk and become a therapeutic target for MetS. For healthy community residents, prospective population-based studies are necessary to investigate mechanisms such as effective lifestyle improvement and other interventions to control WHtR in adults.

## Supporting information

S1 Table Data of subjects. (ODS)
